# Hypoxia induced exosomal circRNA promotes metastasis of Colorectal Cancer via targeting GEF-H1/RhoA axis

**DOI:** 10.7150/thno.44419

**Published:** 2020-07-09

**Authors:** Haiou Yang, Haiyang Zhang, Yuchong Yang, Xinyi Wang, Ting Deng, Rui Liu, Tao Ning, Ming Bai, Hongli Li, Kegan Zhu, Jialu Li, Qian Fan, Guoguang Ying, Yi Ba

**Affiliations:** 1 Tianjin Medical University Cancer Institute and Hospital, National Clinical Research Center for Cancer, Key Laboratory of Cancer Prevention and Therapy, Tianjin's Clinical Research Center for Cancer, Tianjin, 300060, China.; 2 Division of Gastroenterology and Hepatology, Shanghai Institute of Digestive Disease, China.; 3 Key Laboratory of Gastroenterology and Hepatology, Ministry of Health, Shanghai Jiao-Tong University School of Medicine, Renji Hospital, China.

**Keywords:** exosomes, hypoxia, circRNAs, colorectal cancer, cancer metastasis

## Abstract

Hypoxia is one of the important properties of solid tumor. However, oxygen supply within tumors is generally heterogeneous according to the distance from the nearest blood vessel. The discrepancy of metastatic potential exists between hypoxic cancer cells and relatively normoxic cancer cells. But the molecular mechanism remains poorly understood.

**Methods:** Differential expression of circRNAs in plasma exosomes of CRC patients and normal subjects was performed by screening. Exosomes were isolated by ultra-centrifugation and RNA expressions were determined by RT-qPCR. The migratory capacity of cells was performed by high intension imaging, wound healing assay and transwell chamber migration assay.

**Results:** Circ-133 is enriched in the plasma exosomes of CRC patients and increased with the disease progression. Exosomal circ-133 derived from hypoxic cells delivered into normoxic cells and promoted cancer metastasis by acting on miR-133a/GEF-H1/RhoA axis. Meanwhile, animal experiments revealed that knockdown of circ-133 can inhibit tumor metastasis. Circ-133 is expected to be a new biomarker for monitoring tumor progression and might be a novel therapeutic target.

**Conclusions:** Hypoxia-derived exosomal circ-133 transported into normaxic cancer cells and promoted cell migration via miR-133a/GEF-H1/RhoA axis. This study reveals a potential mechanism for that the intra-tumor heterogeneity of oxygen promote cancer progression.

## Introduction

On the basis of the estimated results in 2018, CRC has become the second leading causes of cancer-related death, and the incidence of it has ranked the third for both sexes 1. Early diagnostic rate of CRC has been improved by standard periodic physical examination. But even in developed countries, nearly a quarter of patients are diagnosed at an advanced stage 2. The prognosis of metastatic colorectal cancer (mCRC) is poor and the 5-year survival rate is low 3. For exploring new potential therapeutic targets, it is urgent to further study the mechanism of invasion and metastasis of CRC.

Hypoxic microenvironment is a substantive characteristic of solid tumor which results from rapid proliferation of cancer cells plus abnormal structure and function of tumor blood vessels 4. It is linked with cancer metastasis 5. In low oxygen tensions the Hypoxia-inducible factor 1α (HIF-1α) protein is stabilized and translocates to the nucleus where it modulated the expression of genes which drive adaptation to hypoxic stress 6. Considerable work has been done to characterize the role of HIF-1α in cancer metastasis 7-10. Partial pressure of oxygen attenuates with the distance to the nearest blood vessel. Therefore, cancer cells can be roughly separated into two categories according to the distance from the blood vessels: the relatively normoxic cells and the hypoxic cells 11. The heterogeneity of oxygen supply give rise to different migratory potential between these two groups of cancer cells. In view of the fact that HIF-1α protein will be quickly degraded under aerobic context; it cannot act as a hypoxic-derived signal molecular to directly affect normoxic cancer cells.

Circular RNAs (circRNAs) are a kind of endogenous RNA formed by alternative splicing, and are widely distributed in eukaryotic cells. Owing to its closed ring structure, circRNAs are not easily degraded by endonuclease and are more stable than linear RNA 12. Previous researches have proposed that circRNAs can function as competitive endogenous RNAs (ceRNAs) to sequester its target microRNAs (miRNA) and then diminish the repressive effects on downstream molecules of miRNA 13. Recent studies have confirmed that circRNAs can be enriched in exosomes stably, which are extracellular nanoscale vesicles with a size of 30-100 nm, suggesting that exo-circRNAs is message media that conduct the communication among cancer cells and even can be used as disease biomarker 14, 15.

Metastasis is a multistep complex process that be restrained by an intricate regulation network 16. Cancer cells gain the ability of escaping from primary location is the initial step of invasion-metastasis cascade. E-cadherin is the major mediator of calcium-dependent intercellular adhesion, limits cell motility and establishes cell apical-basal polarity 17, 18. E-cadherin failure to localize to the membrane allows cancer cells to acquire the ability of moving freely. Rho guanine nucleotide exchange factor (GEF-H1, also known as ARHGEF2) is a class of regulatory proteins that promote conversion between active and inactive forms of Rho GTP enzyme by regulating its GDP/GTP exchange 19, 20. Previous studies have demonstrated that GEF-H1/RhoA is involved in the regulation of cytoskeleton components and plays a key role in cancer invasion and metastasis 21-23.

In the case of insufficient oxygen supply, a vast majority of the adaptive reprogramming in cancer cells are driven by HIF-1 protein 11, 24. But it cannot serve as a directly communication mediator between hypoxic cancer cells and normoxic cancer cells. Studies have shown that exosomes are effective carrier of information among cells 25, 26. Meanwhile, researches demonstrated that circRNAs perform epigenetic regulation in tumorigenesis and progression and can be stably wrapped into exosomes to conduct the delivery of transcellular signal 27, 28. At present, it is believed that not only glycolytic pathway but also oxidative phosphorylation is involved in energy metabolism in cancer cells. The imbalance of oxygen nutrition may change the contribution of glycolytic pathway and oxidative phosphorylation pathway to energy generation in cells, thus may resulting in the heterogeneity of the energy reserve between hypoxic cells and normoxic cells 29. Therefore, the group of cancer cells which directly exposed to hypoxic stress may not have optimal metastatic potential. The purpose of this study was to investigate the communication mechanism between hypoxic cancer cells and normoxic cancer cells. We wonder to know whether hypoxic stress can affect the behavior of cancer cells that had a relatively normal oxygen supply.

## Methods and Materials

### Human tissue

CRC tissues and paired adjacent noncancerous tissues were obtained from patients undergoing surgical procedures at the Tianjin Medical University Cancer Institute and Hospital (Tianjin, China). Both the tumor tissues and noncancerous tissues were histologically confirmed. Written consent was provided by all of the patients, and all aspects of this study were approved by the Ethics Committee of Tianjin Medical University Cancer Institute and Hospital. Tissue fragments were immediately frozen in liquid nitrogen at the time of surgery and stored at -80 °C.

### Animals

Female nude mice (BALB/c-nu, 6-8 weeks) were purchased from the Model Animal Center of Nanjing University, fed in an SPF (specific pathogen-free) animal facility. All of the experimental procedures were performed in accordance with protocols approved by the Institutional Animal Care and Research Advisory Committee of Tianjin Medical University Cancer Institute and Hospital.

### Cell culture

Human colorectal cancer cell lines HCT116 and SW480 were purchased from the Shanghai Institute of Cell Biology of the Chinese Academy of Sciences (Shanghai, China). HCT116 were cultured in 1640 (Gibco, USA), and SW480 were cultured in L15 medium (Gibco, USA). Both of the basal culture media above were supplemented with 10% fetal bovine serum (FBS, Gibco, USA) and 1% penicillin/streptomycin. Cells were incubated in a humidified incubator at 37 °C with 5% CO_2_. Hypoxia studies were carried out at 1% oxygen.

### Isolation of exosomes from medium and plasma

Exosomes were isolated from cell culture medium and plasma by sequential differential centrifugation, according to previous publications 30. The cell culture and plasma were centrifuged at 300 g and 3000 g to remove cells and other debris, and the supernatant was centrifuged at 10000 g to remove shedding vesicles and other vesicles with larger sizes. Finally, the supernatant was centrifuged at 110,000 g for 70 min (all steps were performed at 4 °C). Exosomes were harvested from the pellet and resuspended in PBS.

### Transmission electron microscopy assay

For conventional transmission electron microscopy, the exosome pellet was placed in a droplet of 2.5% glutaraldehyde and fixed overnight at 4 °C. Then, the sample was post fixed in 1% osmium tetroxide at room temperature (RT), embedded in 10% gelatin, fixed in glutaraldehyde at 4 °C, and cut into several blocks (<1 mm^3^). The samples were dehydrated in increasing concentrations of alcohol (30, 50, 70, 90, 95 and 100%; ×3). Pure alcohol was then exchanged for propylene oxide, and specimens were infiltrated with increasing concentrations of Quetol-812 epoxy resin mixed with propylene oxide. Samples were embedded in pure, fresh Quetol-812 epoxy resin. Ultrathin sections (100 nm) were cut using a Leica UC6 ultramicrotome and were poststained with uranyl acetate and lead citrate for 5 min at RT before observation via a FEI Tecnai T20 transmission electron microscope operated at 120 kV.

### PKH26 staining for exosomes

PKH26 Red Fluorescent Cell Linker Kits (Sigma) were used for lipid bilayer labeling. Exosomes were first resuspended in 100 µl of Diluent C. A dye solution (4 ×10^-6^ M) was prepared by adding 0.4 µl of PKH26 ethanolic dye solution to 100 µl of Diluent C. The 100 µl of exosome suspension was then mixed with the 100 µl of dye solution by pipetting. After incubating the cell/dye suspension for 1-5 min with periodic mixing, the staining was stopped by adding 200 µl of serum and incubating for 1 minute. The stained exosomes were finally washed twice with 1× PBS and were resuspended in a fresh sterile conical polypropylene tube.

### Cell transfection

HCT116 and SW480 were seeded into 6-well plates and transfected using Lipofectamine 2000 (Invitrogen) and Opti-MEM (Gibco, USA) according to the manufacturers' instructions. For circ-133/miRNA upregulation and downregulation, a 100 pmol dose of circ-133-plasmid, si-circ-133, miR-133a mimics, inhibitors and negative control (NC) were used. In addition, HCT116 and SW480 were harvested 24 h or 48 h after transfection to isolate total RNA or total cell lysate. HCT116 and SW480 cells were cultured in 100 mm dishes and were transfected with si-circ-133 and NC (normal control), and the culture medium was replaced with 1640/L15 (Gibco, USA) with exosome-free FBS (Gibco, USA) for the isolation of exosomes.

### RNA isolation and quantitative RT-PCR

Total RNA was extracted from the cultured cells and tissues using TRIzol reagent (Invitrogen) according to the manufacturer's protocols. We adopted TaqMan microRNA probes (Applied Biosystems, Foster City, CA) to quantitate miRNA. After the reactions were complete, the cycle threshold (CT) data were determined using fixed threshold settings, and the mean CT values were determined from triplicate PCRs. We used the formula to calculate the relative quantities of target genes. U6 snRNA was used as an internal control for the miRNAs; β-actin, for the circ-133 and GEF-H1 mRNA levels. The β-actin, GEF-H1 and circ-133 primers were designed as follows:

5'-TCTATCCTGTGCTCTACCCCA-3' (circ-133, Forward primer);

5'-TGAGAAGACAAGGTGGCCGAG-3' (circ -133, Reverse primer);

5'-TCCCTCATTGACGAAGCAGA-3' (GEF-H1, Forward primer);

5'-GGTGCAGCTCTGTCTGGATT-3' (GEF-H1, Reverse primer);

5'-TCGAGGTGGATGGAAAGCAG-3' (RhoA, Forward primer);

5'-CACAAGACAAGGCACCCAGA-3' (RhoA, Reverse primer);

5'-GGCTGTGCTATCCCTGTACG-3' (β-actin, Forward primer);

5'-CTTGATCTTCATTGTGCTGGGTG-3' (β-actin, Reverse primer).

### Western blot analysis

The expression of protein was assessed by western blot analysis, and its expression in the samples was normalized to β-actin expression. Cells and tissues were lysed in RIPA buffer with freshly added protease inhibitor cocktail. Total lysates were separated on SDS-PAGE gels and transferred to PVDF membranes (Millipore). The immunoblots were blocked with 5% BSA at room temperature for 1 h and incubated at 4 °C overnight with anti-HIF-1α (1:200, Santa Cruz), anti-GEF-H1 (1:1000, Cell Signaling), anti-RhoA (1:500, Immunoway), anti-E-cadherin (1:500, Santa Cruz), anti- β-actin (1:3000, Santa Cruz), anti-CD63 (1:2000, Abcam), and anti-TSG101 (1:1000, Santa Cruz). After incubation with the secondary antibody, the membranes were visualized with an enhanced chemiluminescence system kit (Millipore, USA) according to the manufacturer's protocol.

### The miRNA Target Prediction and Luciferase Reporter Assay

The miRNA target prediction and analysis were performed with the algorithms from TargetScan (http://www.targetscan.org/), PicTar (http://pictar.mdc-berlin.de/) and miRanda (http://www.microrna.org/). The reporter plasmid p-MIR-circ-133, p-MIR-GEF-H1 and p-MIR-RhoA was designed by Genscript (Nanjing, China). For the luciferase reporter assays, 2 mg of firefly luciferase reporter plasmid; 2 mg of β-galactosidase vector; and equal doses (200 pmol) of mimics, inhibitors, or scrambled NC RNA were transfected into cells. The β-galactosidase vector was used as a transfection control. At 24 h after transfection, cells were analyzed using a Dual Luciferase Assay Kit (Promega).

### Cell migration assay

High intension imaging (PerkinElmer, PE operetta CLS, USA), wound healing assay and Transwell chamber (Corning, USA) migration assay was used to determine the migratory capacity of SW480 and HCT116. For the wound healing test, cells were seeded in 6-well plates. In addition, 24 h later, each well was scraped with a 20 µl pipette tip to create 2 linear regions devoid of cells, and medium without FBS was added. Then, at the time points of 0 h, 12 h and 24 h, cells were observed and photographs were taken. For the Transwell chamber test, the pretreated cells were transferred into the upper chamber in 200 µl of serum-free growth medium (10^5^ cells per 8.0 µm pore size polycarbonate membrane insert). In addition, 500 µl of complete media was added to the lower compartment. After incubation for 18-24 h, non-migrated cells on the upper surface of the insert were removed gently by cotton swabs, and cells that migrated to the bottom of the membrane were fixed and stained using a Three-Step Stain Set (Thermo). The number of invaded cells was counted under a light microscope. To minimize the bias, cells in five randomly selected fields at a 200× magnification were counted to calculate the average cell number.

### Immunofluorescence

Tissue double-labeled immunofluorescence was performed in paraffin-embedded colorectal cancer and its adjacent tissues. Paraffin sections were baked in an oven at 70 °C for 1-2h, then soaked in xylene and alcohol in turn to dewax, and placed in boiling citrate repair solution for antigen repair, 0.2%TritonX-100 for permeation, 5%BSA for antigen closed, then incubated overnight with a primary antibody mixture. After washing with PBS, the secondary antibody was incubated. After sealing, it was observed under a confocal microscope. The cells to be detected by immunofluorescence were seed in a 12-well plate and grown on coverslips, after fixing and incubating the antibodies, it was also observed by confocal microscopy.

### Determination of RhoA-GTP content

The content of RhoA-GTP were performed by Human RhoA-GTP ELISA KIT (shanghai FANKEL Industrial Co., Ltd.). Cell suspension diluted with PBS to a concentration of 1 million/ml. After repeated freezing and thawing, the cell rupture and release of intracellular components. Centrifuge at 2000-3000 rpm for 20 minutes at RT, carefully collected the supernatant. Then follow the instructions for subsequent enzyme labeling, incubation and color rendering. Finally, OD value at 450 nm was analyzed by microplate reader.

### RNA-RNA pull-down

Dynabeads™ Streptavidin Trial Kit (Invitrogen, USA) was used to complete the experiment. Biotinylated miR-133a and its corresponding negative controls were transfected into 293T cells respectively. After 24 hours of culture, the cells were lysed. And the cell lysates were co-incubated with the streptavidin beads. The beads are eluted with the aid of the DynaMag™ Magnet. Finally, PCR was performed for eluting complexes to analyze the abundance of circ-133, GEF-H1 mRNA and RhoA mRNA.

### Establishment of tumor xenografts in mice

The lentiviral expression plasmids were bought from Shanghai Genechem Co. Ltd. Puromycin (Sigma-Aldrich, USA) was used to successfully infect HCT116 cells and obtain stable high expression of GEF-H1, low expression of GEF-H1 and control HCT116 cells. Then, those cells were inoculated subcutaneously into SCID mice (3 × 10^6^ cells in 0.2 mL PBS per mouse, 6 mice per group). After a week, Exosomal-OE.circ-133 or Exosomal-KD.circ-133 were injected into caudal vein every three days. Mice were sacrificed 28 days after injection to remove the xenografted tumors, and the volumes and weights of the tumors were recorded. The tumor was divided into two parts; one part was used for protein and total RNA extraction, and the remaining tissue was used for immunohistochemical staining for GEF-H1 and E-cadherin. CTCs were detected by were detected by flow cytometry.

### Statistical analysis

The results are presented as the average of at least three experiments, each performed in triplicate, with standard errors. Data were described with median values ± SME and analyzed by using Student's t-test for 2-group comparisons. Differences were considered statistically significant at P<0.05. In this study, '*' indicates P<0.05,' '**' indicates P<0.01, and '***' indicates P<0.001.

## Results

### Heterogeneity of oxygen supply affects the distribution of E-cadherin across the membrane

**Figure 1:**
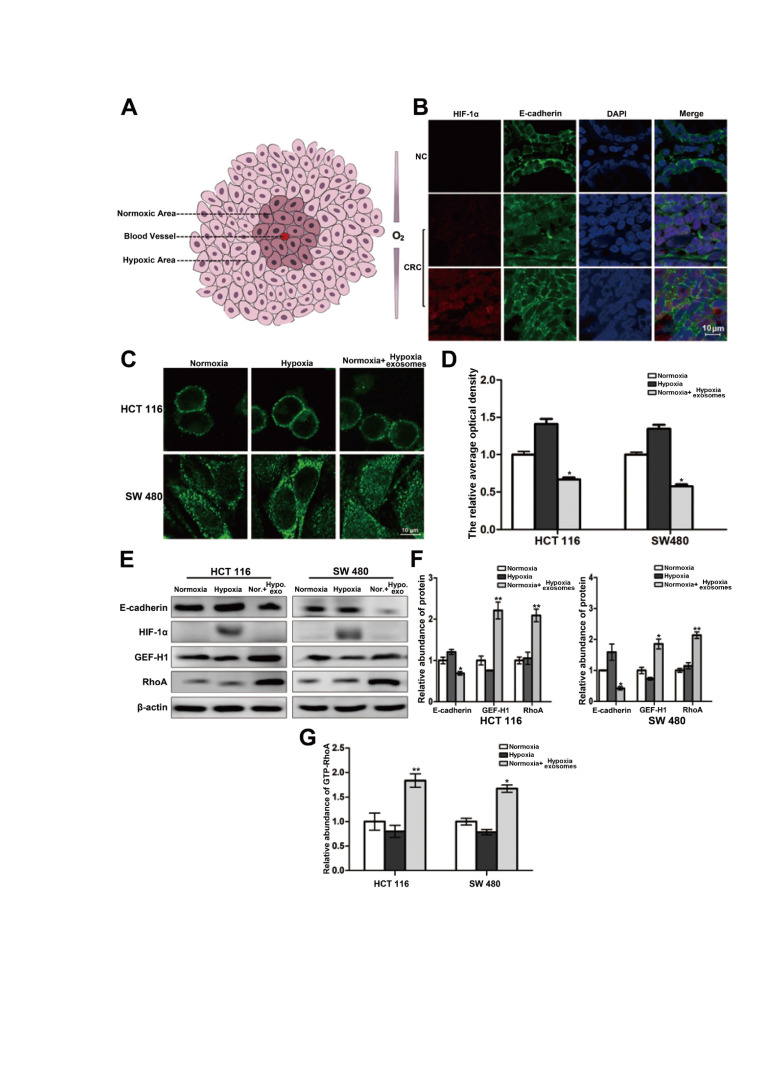
**Heterogeneity of oxygen supply affects the membrane distribution of E-cadherin in CRC.** (**A**) Briefly graphical representation of intra-tumoral oxygen heterogeneity. (**B**) Double-labeled immunofluorescence was performed in CRC tissue and its corresponding paracancer tissue, HIF-1α was labeled with Alexa Fluor^®^ 594, E-cadherin was labeled with Alexa Fluor^®^ 488, (n=5). (**C**) Directly evidence of the effect of differences in oxygen supply on E-cadherin membrane distribution in HCT116 and SW480 cells, (n=3). (**D**) Quantitative analysis of (C). (**E**) Associated proteins expression in each treatment groups (normoxic culture group, hypoxic culture group and hypoxic cell exosomes treated normoxic culture group), (n=3). (**F**) Quantitative analysis of (E). (**G**) Enzyme-linked immunosorbent assay was performed to detect the content of GTP-RhoA in each treatment group (normoxic culture group, hypoxic culture group and hypoxic cell exosomes treated normoxic culture group), (n=3).

The interior of a rapidly growing solid tumor is hypoxic. But the oxygen supply to cancer cells varies depending on the distance to the feeding arteriole. Therefore, cancer cells can be roughly divided into two groups, namely hypoxic cancer cells and normoxic cancer cells (Figure 1A). The results of double-labeled immunofluorescence in paraffin sections of colorectal cancer and its para-carcinoma tissues showed that compared with the para-carcinoma normal cells, the distribution of E-cadherin across the cell membrane of colorectal cancer generally trended to decrease. Moreover, the E-cadherin distribution across the membrane of normoxic cancer cells is less than that of hypoxic cancer cells (Figure 1B). It is suggested that the difference of oxygen nutrient status is closely related to the metastasis potential of cancer cells. And as shown in Figure 1C-1D (three treatment groups: normoxic culture group, hypoxic culture group and hypoxic cell exosomes treated normoxic culture group), the E-cadherin across the membrane of normoxic cells which coculture with hypoxic exosome reduced. Moreover, the capacity of cell migration increased after incubating by hypoxic exosome (Figure S1A-S1B). Hypoxic-derived exosomes may indeed carry pro-metastasis signals to normoxic cancer cells.

The connection of the intracellular domain of E-cadherin to the actin cytoskeleton is important for maintaining colonic epithelial homeostasis. Tight junction-associated protein GEF-H1/RhoA coordinates the remodeling of the cytoskeleton and is engaged in cancer metastasis including colorectal cancer. Therefore, we detected whether GEF-H1/RhoA was changed during the above hypoxic stimulation. After verification, it was found that the expression of GEF-H1/RhoA in the hypoxic exosomes-treated normoxic group was overexpressed, and RhoA activity is correspondingly increased downstream (Figure 1E-1G). These results revealed the normoxic cells stimulated by hypoxic exosomes are more likely to metastasize.

### MiR-133a directly targets GEF-H1 and RhoA

**Figure 2:**
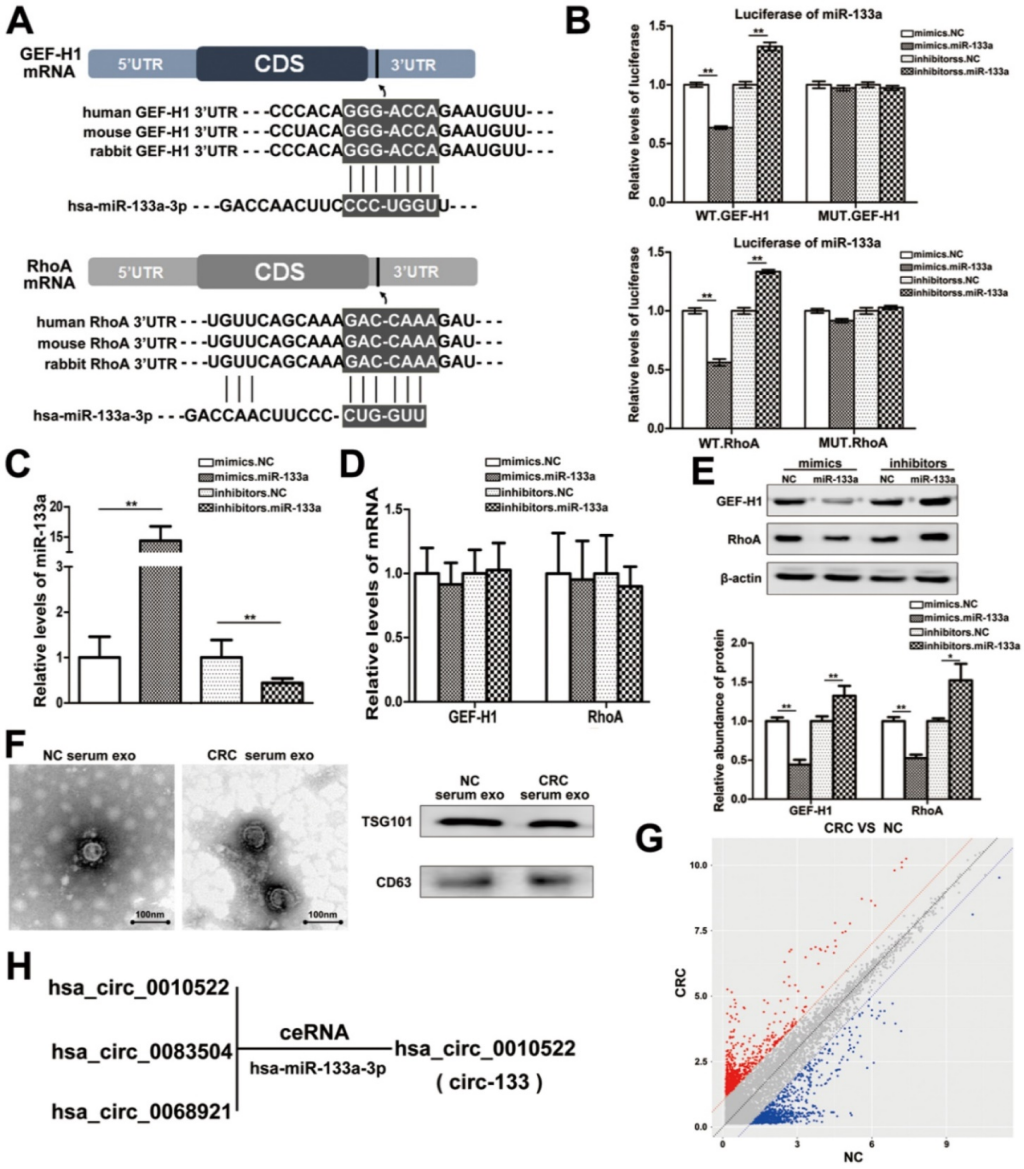
**MiR-133a is directly targeted at GEF-H1/RhoA, and is adsorbed by circ-133.** (**A**) Predicted binding sites of miR-133a within the 3'UTR of GEF-H1 mRNA and RhoA mRNA. (**B**) Direct recognition of GEF-H1 3'UTR and RhoA 3'UTR by miR-133a, (n=3). (**C**) Quantitative RT-PCR analysis of the relative miR-133a levels in each treatment group (n=3). (**D**) Quantitative RT-PCR analysis of the relative GEF-H1 mRNA and RhoA mRNA levels in each treatment group, (n=3). (**E**) Western blot analysis of GEF-H1 and RhoA expression in each treatment groups, (n=3). (**F**) Electron microscope scanning of exosomes isolated from CRC plasma and NC plasma. And western blot analysis of exosome-enriched protein CD63 and key proteins for miRNA function, TSG101 (n=3). (**G**) Preliminary screening of CRC-specific plasma exosomal circRNAs (n = 10). (H) Circ-133 (has_circ_0010522) can directly adsorb miR-133a.

MiRNAs are key components of non-coding RNA that mediate the epigenetic regulation of cancer, and have been extensively studied. The prediction of bioinformatics analysis revealed that the 3′-UTR of both GEF-H1 mRNA and RhoA mRNA can be directly targeted by miR-133a in a highly conserved manner among species (Figure 2A). Luciferase assay was performed to verify the above target relationship, and showed that the relative luciferase activity was clearly inhibited when miR-133a mimics were co-transfected with the luciferase reporters, while the relative luciferase activity was noticeably increased by miR-133a inhibitors (Figure 2B). Additionally, the intracellular abundance miR-133a of cells has also been tested to verify the transfection effect (Figure 2C), while the mRNA content remained unchanged (Figure 2D). Subsequently, the contents of GEF-H1 and RhoA proteins were detected (Figure 2E), which further confirmed that miR-133a negatively regulated the expression of GEF-H1 and RhoA at the post-transcriptional level.

Studies have confirmed that miR-133a is indeed involved in the regulation of colorectal cancer via targeting on a variety of different genes. Then, we supposed that miR-133a could affect the metastatic potential of CRC under hypoxic stimulation by targeting GEF-H1/RhoA. And miR-133a was associated with cancer metastasis (Figure S1C-S1D).

### The direct interaction between circ-133 and miR-133a

**Figure 3:**
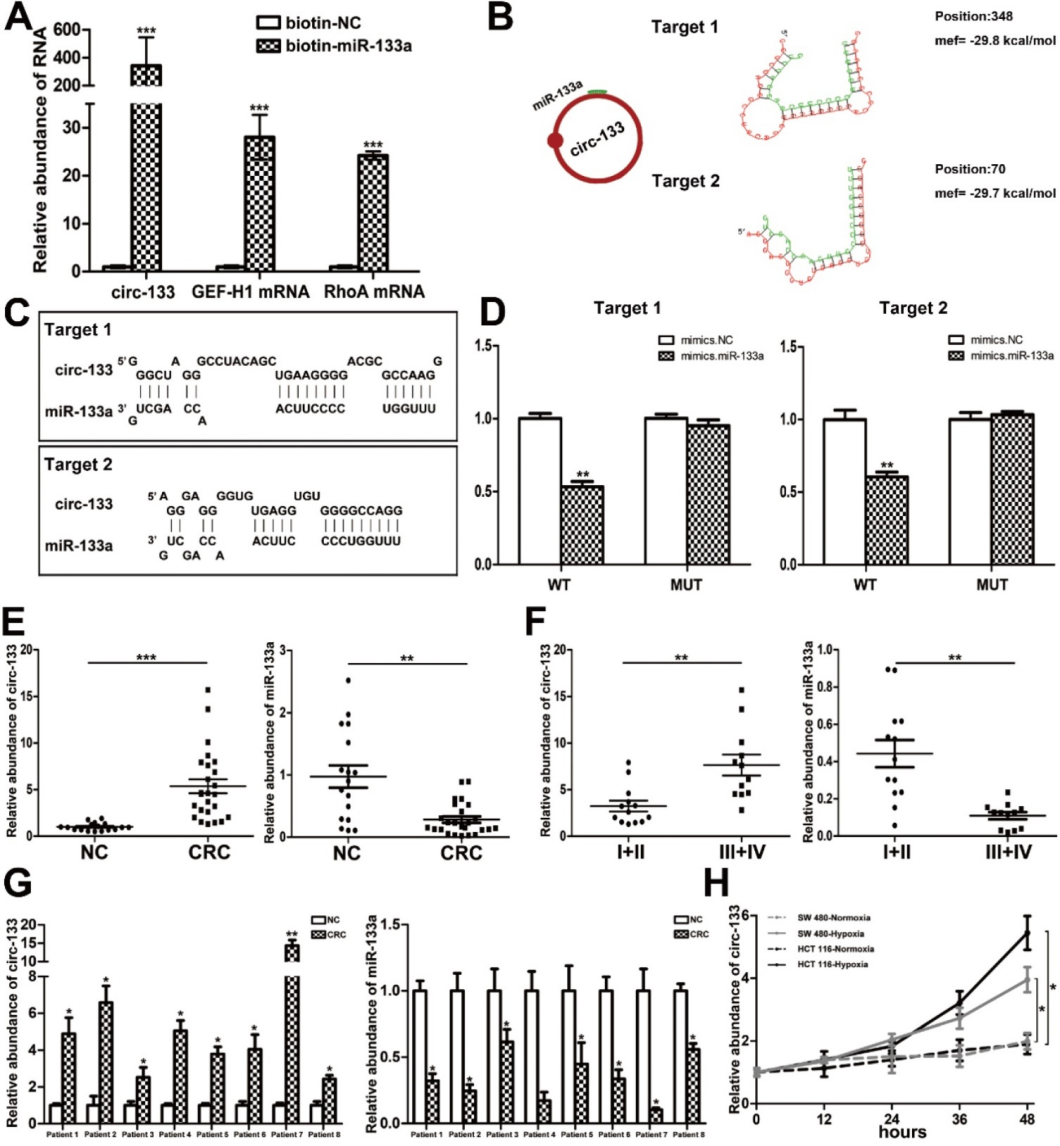
** The direct interaction between circ-133 and miR-133.** (**A**) HEK293T were transfected with biotin-NC and biotin-miR-133a, respectively. And then RNA pull-down was performed to detect the relative level of circ-133, GEF-H1 mRNA and RhoA mRNA, (n=3). (**B**) Graphical representation for two of the binding sites between miR-133a and circ-133. (**C**) The two prediction binding sites of miR-133a and circ-133. (**D**) Direct recognition of circ-133 by miR-133a(n=3). (**E**) The relative level of circ-133 and miR-133a between CRC plasma and NC plasma, (n=3). (**F**) The relative level of circ-133 and miR-133a between I+II and III+IV CRC plasma, (n=3). (**G**) Circ-133 and miR-133a expression in CRC and its corresponding paracancer tissue, (n=3). (**H**) Circ- 133 were gradually accumulated in hypoxic exosomes, (n=3).

CircRNAs are more stable and not easily be degraded depend on their closed loop structure with no 5′ cap or 3′ poly(A) tail. Recent studies have demonstrated that it can be secreted into the extracellular environment by exosomes to mediate intercellular communication. We isolated plasma exosomes from CRC patients and normal subjects, and these exosomes were firstly morphologically identified by transmission electron microscopy, and the exosomal markers Tsg101 and CD63 were also detected (Figure 2F). As is shown in Figure 2G, a panel of exosomal circRNAs in plasma that are differentially expressed between CRC (n=10) and NC (n=10). And as is shown in Figure 2H, three of the significantly up-regulated circRNAs (fold change > 4) can adsorb miR-133a, as follows: has_circ_0010522, has_circ_0083504 and has_circ_0068921, among which has_circ_0010522 (hereinafter referred to as circ-133) had the highest upregulation abundance and was predicted to contain at least 10 miR-133a binding sites. RNA-RNA pull-down assay also confirmed the targeting effect of miR-133a on GEF-H1 mRNA and RhoA mRNA. Meanwhile, circ-133 captured by miR-133a-biotin was significantly increased compared with the NC-biotin (Figure 3A). We selected two of the binding sites of circ-133 and miR-133a (Figure 3B-3C), and performed luciferase assay for the above binding sites, further confirming the interaction between circ-133 and miR-133a (Figure 3D).

### The expression of circ-133 is up-regulated in CRC and is associated with hypoxia

To determine the expression of circ-133, qRT-PCR was performed in 17 normal subjects and 25 CRC patients (Figure S2A). Plasma exosomes were isolated and its RNA was extracted for detection. As shown in Figure 3E and Figure S2B, compared with the normal subject group, the amount of circ-133 in the plasma exosomes of CRC patients was significantly increased, while the amount of miR-133a was decreased. Malignant tumors are classified into stage I, II, III and IV according Tumor-Node-Metastasis (TNM) classification. Comprehensive analysis showed that as the disease progressed, circ-133 expression was gradually elevated while the level of miR-133a reduced (Figure 3F, Figure S2B). In addition, compared with the corresponding paracancer tissues, it was also found that circ-133 was overexpressed in the CRC tissues. (Figure 3G).

Circ-133 expression was higher in CRC cell lines than in normal colonic epithelium cells NCM 460 (Figure S2C). And then we selected cell lines SW480 and HCT116 that showed a moderate level of circ-133 expression for *in vitro* experiments. SW480 and HCT116 were cultured under hypoxic and normoxic conditions respectively. Culture medium was collected and exosomes were extracted to detect the level of circ-133. The results showed that exosomes derived from hypoxic cells were rich in circ-133, which gradually increased with the prolongation of hypoxic time (Figure 3H).

### Hypoxic derived exosomal circ-133 promotes tumor metastasis through targeting of GEF-H1/RhoA

**Figure 4:**
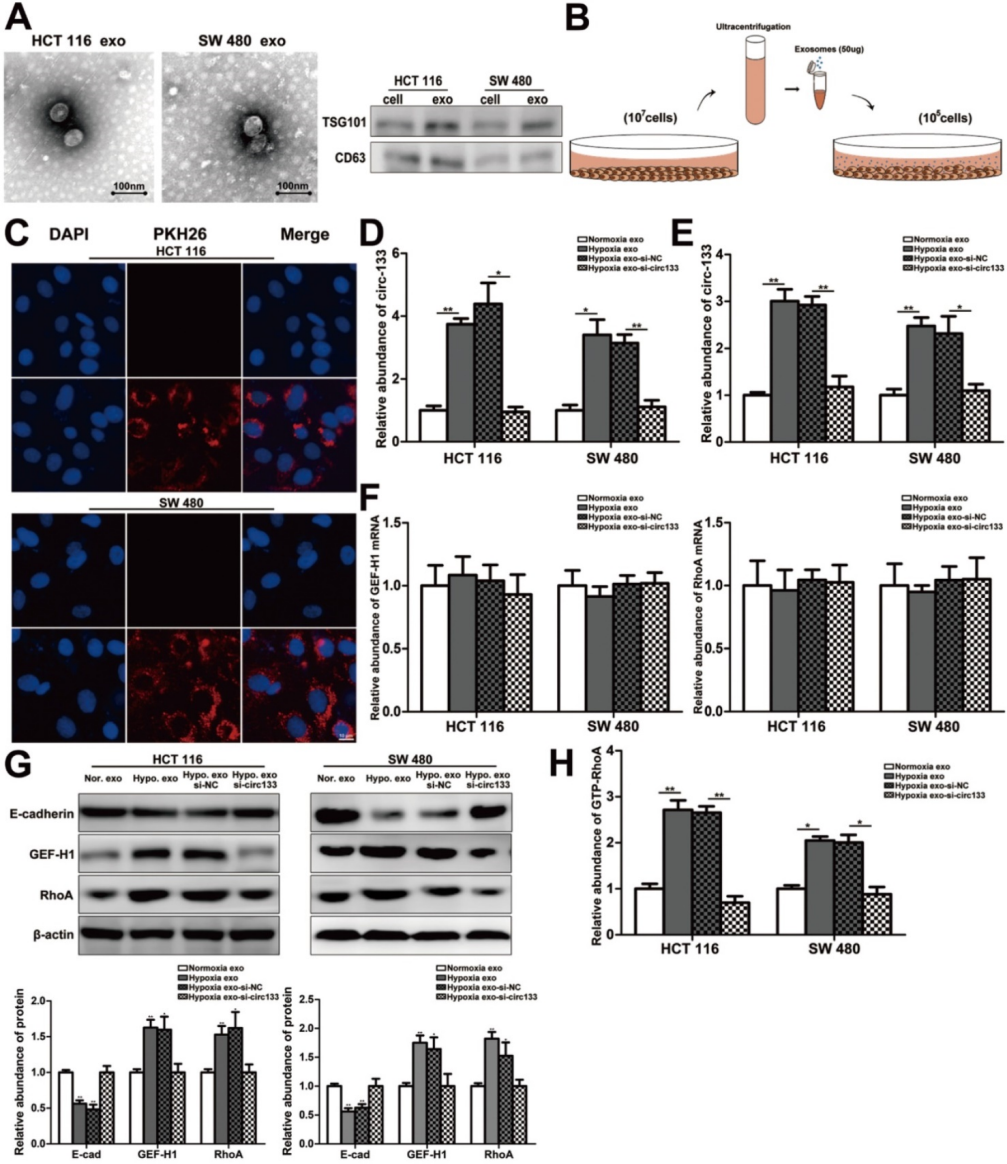
**Hypoxic exosomes incubate normoxic CRC cells.** (**A**) Electron microscope scanning of exosomes derived from HCT116 and SW480 cells. And western blot analysis of exosome-enriched protein CD63 and key proteins for miRNA function, TSG101 (n=3). (**B**) Schematic description of the experimental design. (**C**) Confocal microscopy image of the internalization of fluorescently labelled exosomes in HCT116 and SW480 cells. (**D**) Quantitative RT-PCR analysis of the content of circ-133 in normoxia exosome, hypoxia exosome, hypoxia si-NC exosomes and hypoxia si-circ-133 exosomes, (n=3). (**E**)The content of circ-133 in HCT116 and SW480 cells that pretreated with normoxia exosome, hypoxia exosome, hypoxia si-NC exosomes and hypoxia si-circ-133 exosomes, respectively, (n=3). (**F**) The level of GEF-H1 mRNA and RhoA mRNA in each treatment group (treated with normoxia exosome, hypoxia exosome, hypoxia si-NC exosomes and hypoxia si-circ-133 exosomes, respectively), (n=3). (**G**) Associated proteins expression in each treatment groups, (n=3). (**H**) The abundance of GTP-RhoA in each treatment group, (n=3).

In our study, exosomes were isolated by sequential differential centrifugation, morphological identification by transmission electron microscopy, and specific markers detection by western blotting (Figure 4A). As shown in Figure 4B, SW 480 and HCT 116 cells in normoxic culture were treated with the extracted exosomes. The PKH26 staining of exosomes confirmed that they can successful fusion into recipient cells (Figure 4C). In this study, exosomes with different abundance of circ-133 were obtained by transfection with their corresponding donor cells, including four treatment groups: normoxic exosome, hypoxia exosome, hypoxia si-NC exosomes and hypoxia si-circ-133 exosomes. The level of circ-133 in exosomes in each group has been verified (Figure 4D). Meanwhile, circ-133 in cells incubated with hypoxic exosomes was increased, while that in the hypoxia si-circ-133 exosomes treated group decreased correspondingly (Figure 4E). And the level of GEF-H1 mRNA and RhoA mRNA kept almost constant (Figure 4F). The results of western blotting revealed that the E-cadherin in the hypoxia exosomes treated group was reduced, while the expression of GEF-H1 and RhoA were increased. However, when circ-133 in hypoxic exosomes was counteracted, the above changes were weakened or even disappeared (Figure 4G). In addition, the relative abundance of RhoA-GTP was detected, confirming that RhoA activation was elevated in the hypoxia exosomes treated group and was hindered in the hypoxia si-circ-133 exosomes co-incubated group (Figure 4H).

**Figure 5:**
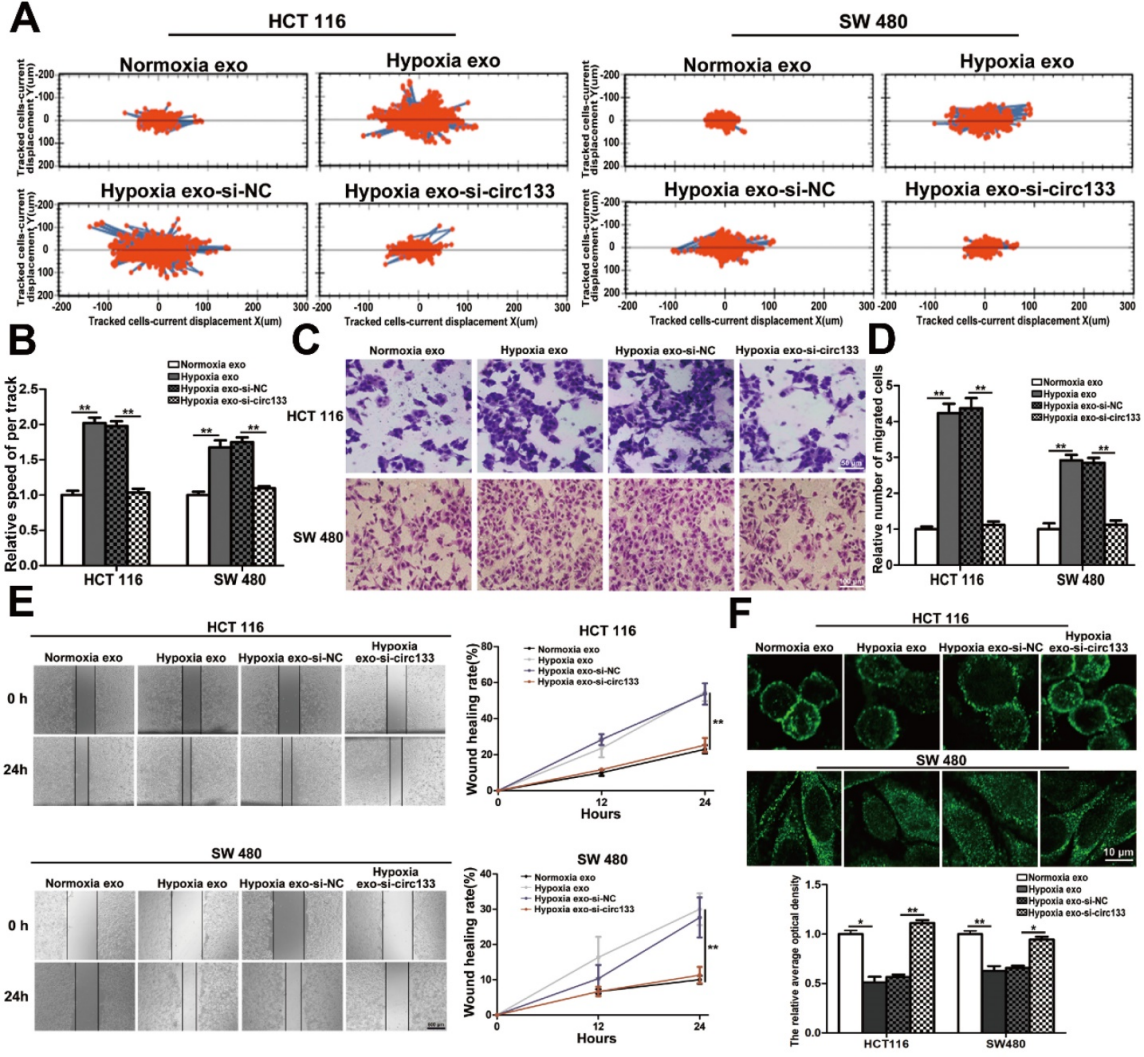
**Hypoxic exosomal circ-133 promotes tumor metastasis through targeting of GEF-H1/RhoA.** (**A**) High intension imaging cell analysis revealed that hypoxic derived exosomes enriched circ-133 increase CRC cells migration, (n=3). (**B**) Quantitative analysis of A, (n=3). (**C**) Validation of hypoxic exosomes derived circ-133 enhance the migration ability of CRC cells by transwell assays, (n=3). (**D**) Quantitative analysis of C, (n=3). (**E**) Wound healing assay demonstrated that hypoxic exosomes promote CRC cell migration, (n=3). (F) Immunofluorescence assay was used to detect E-cadherin membrane distribution in each treatment group, (n=3).

In this study, high intension imaging cell analysis, transwell assay and wound healing test were combined to analyze changes in cell migration capacity. As shown in Figure 5A and 5B, when the cells were treated with hypoxic exosomes, their motor capacity enhanced, but after the elimination of circ-133 from the hypoxic exosomes, this phenomenon of promoting cell movement disappeared. Similarly, after co-incubating with the hypoxic exosomes, the number of cells passing through the compartment increased and scratch healing accelerated, whereas the hypoxic si-circ-133 exosome treatment group did the opposite (Figure 5C-5E). Immunofluorescence further confirmed that hypoxic exosomes-derived circ-133 reduced the distribution of E-cadherin on the cell membrane (Figure 5F).

### Directly validation of circ-133 promotes cell migration via the miR-133a/GEF-H1 /RhoA axis

**Figure 6:**
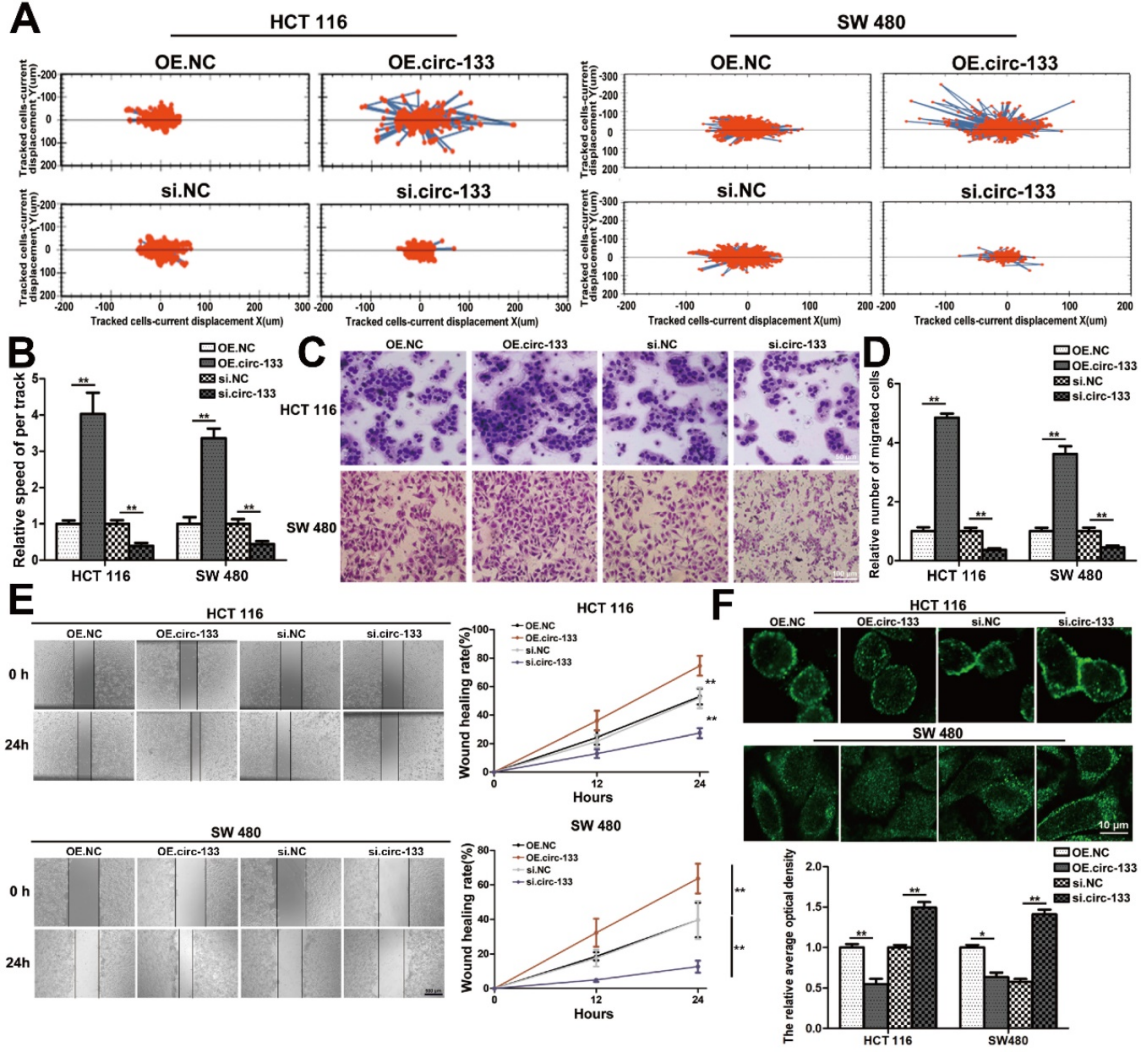
**Validation of circ-133 promotes the migration ability of normoxic CRC cells directly.** OE circ-133, OE NC, si-circ-133 and si-NC were transfected into CRC cells, respectively. (**A**) High intension imaging cell analysis showed that circ-133 promotes the migration of normoxic CRC cells (n=3). (**B**) Quantitative analysis of A, (n=3). (**C**) Validation of circ-133 mediated normoxic CRC cell migration by transwell assay (n=3). (**D**) Quantitative analysis of C, (n=3). (**E**) Wound healing assay demonstrated circ-133 enhance the migration ability of normoxic CRC cells (n=3). (**F**) Immunofluorescence assay was used to detect E-cadherin membrane distribution in each treatment group, (n=3).

The abundance of circ-133 in SW480 and HCT116 cells were directly changed by transfection, including four groups: OE. circ-133, OE. NC, si-circ-133 and si-NC. High intension imaging cell analysis showed that the motility of cells in OE. circ-133 group enhanced, while that in si-circ-133 group reduced (Figure 6A-6B). And the result of transwell assay found that the number of cells passing through the compartment increased with the overexpression of circ-133, and decreased with the downregulation of circ-133 (Figure 6C-6D). As shown in Figure 6E, the ability of cell scratch healing was accelerated in the OE. circ-133 group, but weakened in the si-circ-133 group. Cell immunofluorescence assay also showed that the level of circ-133 in cells was negatively correlated with the E-cadherin membrane distribution of normoxic cells (Figure 6F).

**Figure 7:**
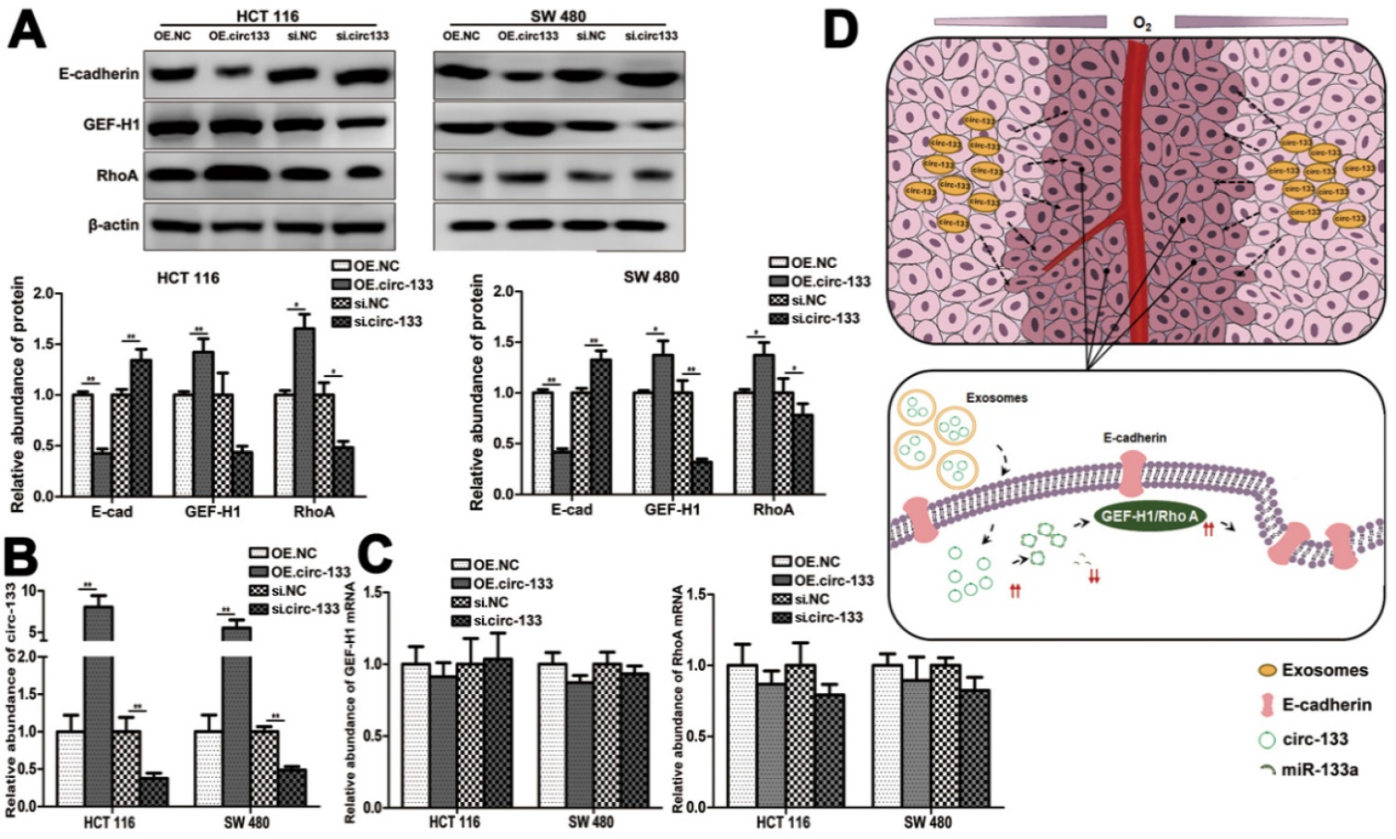
** A proposed mechanistic model about this study.** (**A**) Associated proteins expression in each treatment groups (OE circ-133, OE NC, si-circ-133 and si-NC), (n=3). (**B**) The expression of circ-133 in each group, (n=3). (**C**) The level of GEF-H1 mRNA and RhoA mRNA in each treatment group, (n=3). (**D**) A proposed mechanistic model about this study.

Meanwhile, the expression of GEF-H1 and RhoA was up-regulated and E-cadherin was reduced in cells of the OE. circ-133 group, while that of the si-circ-133 group was reversed (Figure 7A). The results of qRT-PCR confirmed the corresponding changes of circ-133 in the transfected cells (Figure 7B), while the GEF-H1 mRNA and RhoA mRNA remained stably (Figure 7C).

Thus, our hypothesis was confirmed *in vitro*, that hypoxic cells-derived exosomal circ-133 were transported into relative normoxic cells and then targeted GEF-H1/RhoA by adsorbing miR-133a, thereby reducing the distribution of E-cadherin on the membrane, enhancing the migration ability of cancer cells (Figure 7D).

### Hypoxia-driven circ-133/GEF-H1/RhoA axis was further confirmed to promote tumor progression *in vivo*

**Figure 8:**
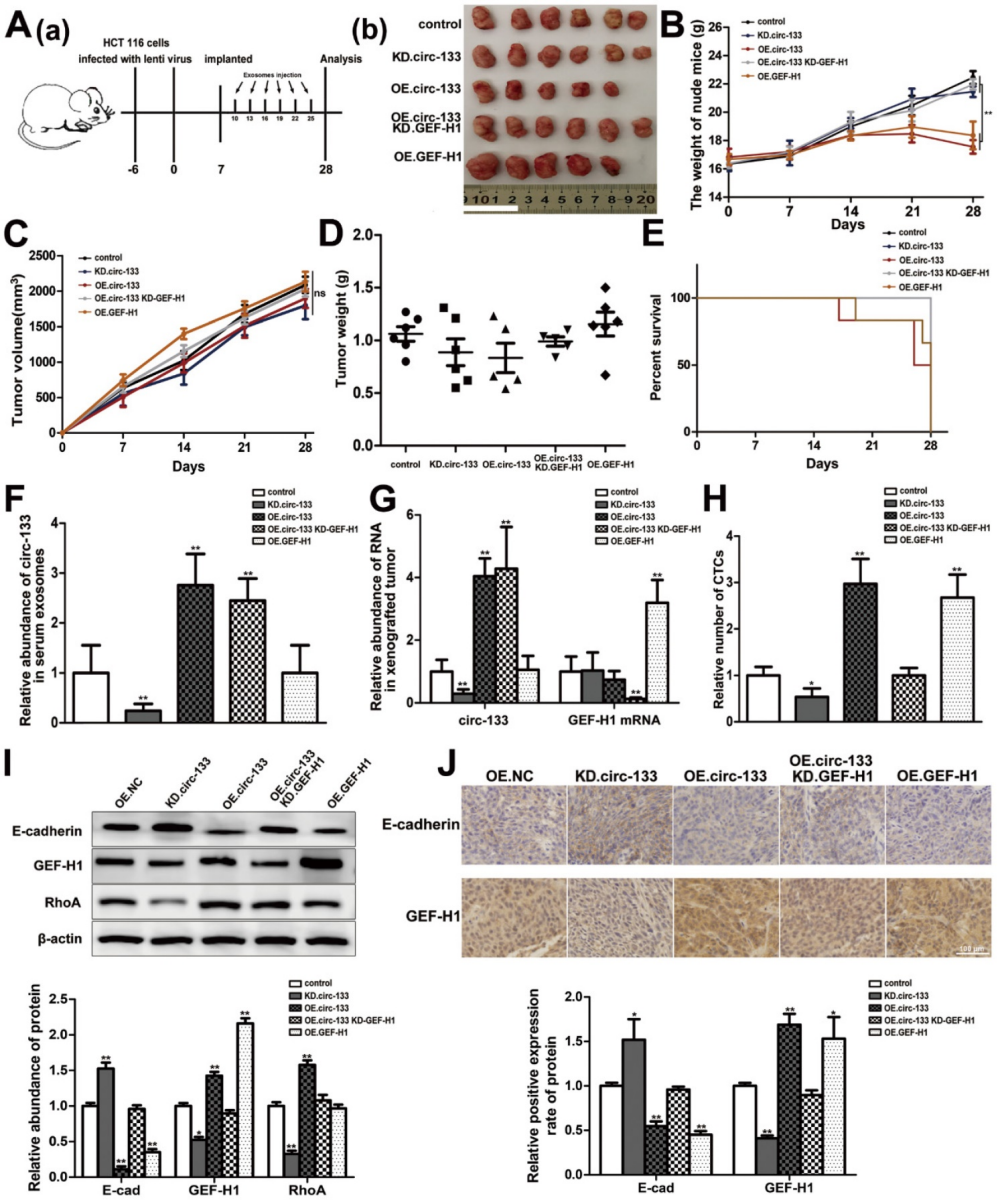
***In vivo* verification for hypoxic exosomes derived circ-133 promotes cancer metastasis.** (**A** (a)) A flow chart depicting the *in vivo* experimental design. (**A** (b)) The morphology of the tumor tissues excised from tumor-implanted mice (n = 6). (**B**) Weight changes during tumor-bearing period in each group (n=6). (**C and D**) Quantitative analysis of xenografted tumor volume and Weight (n=6). (**E**) During the tumor-bearing period, survival of mice in each group. (**F**) The level of circ-133 in exosomes that isolated from plasma of tumor-implanted mice (n=6). (**G**) qRT-PCR analysis of circ-133 and GEF-H1 mRNA in implanted tumors (n=6). (**H**) Relative numbers of CTCs in each group (n=6). (**I**) WB analysis of E-cadherin, GEF-H1 and RhoA in implanted tumors (n=6). (**J**) Immunohistochemically analysis of the paraffin-embedded tumor tissues using E-cadherin and GEF-H1 antibody, respectively (n=6).

The effect of the circ-133/GEF-H1/RhoA axis on the metastatic potential of colorectal cancer was then further validated *in vivo*. Figure 8A(a) is a simplified flowchart of the experiment. HCT116 cells were firstly infected with the corresponding lentivirus to obtain stable high expression of GEF-H1, low expression of GEF-H1 and control cell lines. Then, those cells were inoculated subcutaneously into SCID mice. After a week, Exosomal-OE.circ-133 or Exosomal-KD.circ-133 were injected into caudal vein every three days. After 28 days, subcutaneous grafts were harvested surgically (Figure 8A(b)). During the tumor-bearing period, mice weight and tumor growth were recorded (Figure 8B-8C). And the weight of the transplanted tumor is shown in Figure 8D. However, some mice died during the days of the experiment. In the group of OE.circ-133, one mouse died 17 days after tumor-bearing and two died 26 days after tumor bearing; in OE.GEF-H1 group, one mouse died 19 days after tumor-bearing and one mouse died 27 days after tumor-bearing. By 28 days after the implantation (that is, before the operation to obtain the transplanted tumor), the natural survival of mice in each group was shown in Figure 8E. As shown in Figure 8F-8G, the changes of circ-133 in the plasma exosomes of mice in each group were consistent with those in transplanted tumor tissues. The abundance of circ-133 in the plasma exosomes and xenograft tissues of mice in OE.circ-133 and OE.circ-133- KD-GEF-H1 group increased significantly, while that in KD.circ-133 group decreased. Meanwhile, the CTCs of mice in OE.circ-133 group and OE.GEF-H1 group accelerated significantly, but with the down-regulation of circ-133 expression, the number of CTCs reduced correspondingly (Figure 8H). Changes in protein content in transplanted tumor tissues were detected and it was found that E-cadherin, GEF-H1 and RhoA proteins changed with the variation in circ-133/GEF-H1/RhoA axis function (Figure 8I). Immunohistochemical staining results showed that the distribution of E-cadherin protein on the membrane decreased in OE.circ-133 group, and the distribution recovered when the function of the circ-133/GEF-H1/RhoA axis was inhibited (Figure 8J). To conclude, hypoxic exosomal circ-133 can promote cancer metastasis by GEF-H1/RhoA axis and is expected to be a potential therapeutic target.

## Discussion

CRC is still a serious threat to human health due to its high incidence and mortality. Improvements in physical examination and guidelines have made it possible to curb the development of CRC at an early stage. But a certain percentage of patients are still diagnosed at an advanced stage 2. While there are still few treatment options available for patients with mCRC and new therapeutic targets and drugs are urgently needed.

Microenvironmental hypoxia is an important intrinsic property in the rapidly growing solid tumor 4. Current studies have confirmed that the energy metabolism of cancer cells is not only through the accelerated glycolytic pathway, but also through the oxidative phosphorylation pathway that fully oxidizes glucose 29. The oxygen partial pressure in solid tumor is heterogeneous 11. And oxygen nutrient status is an important factor affecting cancer energy metabolism 31. Therefore, different oxygen supply conditions will result in intercellular heterogeneity of cancer energy metabolism, resulting in differences in energy storage between normoxic cells and hypoxic cells, presenting different metastasis potentials.

The regulation of miRNAs to genes at the post-transcriptional level is not one-to-one, but presents a complex network regulation pattern 32. The involvement of miR-133a in the tumorigenesis and tumor progression by targeting different genes has been reported in previous scientific literature 33. And miR-133a can function as a regulator of cancer metastasis by targeting SOX4, MMP9, etc. 34, 35. This study focused on the regulatory effect of miR-133a on GEF-H1 /RhoA, and tended to add a new branch to its regulatory network.

E-cadherin is a pivotal molecule that mediates the connection between epithelial cells, and its reduced distribution on the membrane is a key initial link for the invasion and metastasis of cancer cells 36. However, in this study, the detailed mechanism of the change in E-cadherin membrane distribution caused by GEF-H1 /RhoA was not explored. We hypothesized that the regulation of cytoskeleton components by GEF-H1 /RhoA might affect the intracellular domain of E-cadherin and its endocytosis. However, the specific mechanism still needs to be further studied.

In this study, a group of differentially expressed circRNAs were screened from the plasma exosomes of CRC patients and normal subjects. circ-133 was selected, which is related to hypoxia and regulates the distribution of E-cadherin protein across cell membrane. Then it was verified both *in vitro* and *in vivo* that exosomal circ-133 derived from hypoxic cells transported into normoxic cells and regulated the E-cadherin membrane distribution, promoting cancer metastasis via miR-133a/GEF-H1/RhoA axis.

Circ-133 was enriched in the plasma exosomes of CRC patients and gradually increased with disease progression, suggesting that exosomal circ-133 is expected to be biomarker for monitoring the progression of CRC. In addition, animal experiments revealed that reducing the level of circ-133 can weaken the metastatic potential of CRC, which is a potential new therapeutic target.

## Conclusions

In summary, our study is the first to explore the role of intra-CRC oxygen supply heterogeneity in promoting tumor metastasis. And the intercellular signaling pathway between hypoxic and normoxic cells mediated by exosomes has been revealed for the first time from the perspective of epigenetic regulation. However, there are many components in exosomes, and the carcinogenic effect of heterogeneous oxygen supply is complex, which still needs further study in the future.

## Supplementary Material

Supplementary figures and tables.

## Abbreviations

MiRNAs: microRNAs
CRC: colorectal cancer
circRNA: circular RNA
ncRNAs: noncoding RNA
ceRNA: competing endogenous RNAs
NC: negative control
OE: overexpression
KD: knock down
qRT-PCR: quantitative reverse transcription-polymerase chain reaction
PBS: phosphate-buffered saline
BSA: bovine serum albumin
RT: reverse transcription
