# Hypoxia induced exosomal circRNA promotes metastasis of Colorectal Cancer via targeting GEF-H1/RhoA axis: Erratum

**DOI:** 10.7150/thno.71495

**Published:** 2022-03-06

**Authors:** Haiou Yang, Haiyang Zhang, Yuchong Yang, Xinyi Wang, Ting Deng, Rui Liu, Tao Ning, Ming Bai, Hongli Li, Kegan Zhu, Jialu Li, Qian Fan, Guoguang Ying, Yi Ba

**Affiliations:** 1 Tianjin Medical University Cancer Institute and Hospital, National Clinical Research Center for Cancer, Key Laboratory of Cancer Prevention and Therapy, Tianjin's Clinical Research Center for Cancer, Tianjin, 300060, China.; 2 Division of Gastroenterology and Hepatology, Shanghai Institute of Digestive Disease, China.; 3 Key Laboratory of Gastroenterology and Hepatology, Ministry of Health, Shanghai Jiao-Tong University School of Medicine, Renji Hospital, China.

We regret that the original version of our paper 1 unfortunately contained some unintentional mistakes in Figure 8J and Figure S1B,1D. Incorrect images were included in the process of assembling these Figures. We apologize for any inconvenience that these errors may have caused. The correct figures are shown below. This image substitution does not affect any results and conclusions of our paper.

## Figures and Tables

**Figure 1:**
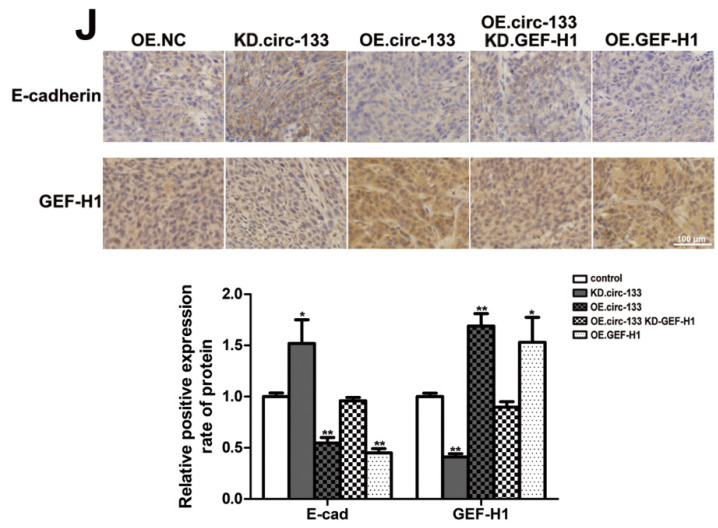
Corrected image for original Figure 8J.

**Figure 2:**
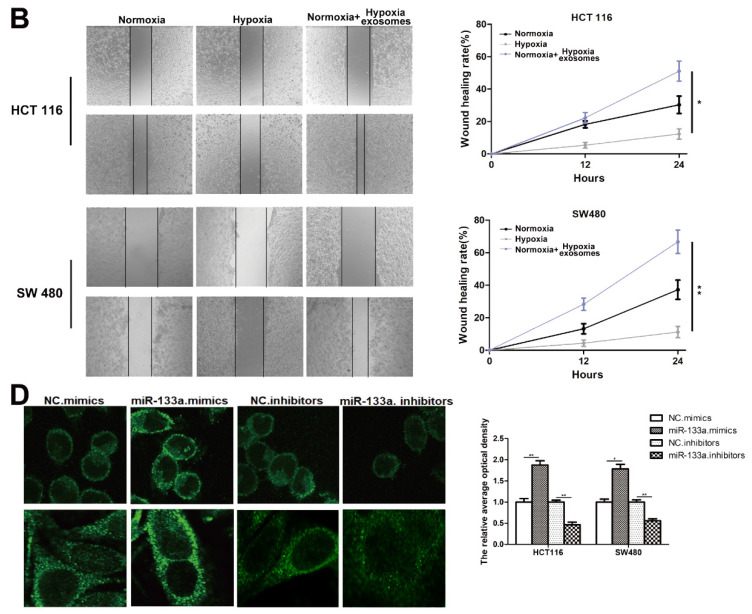
Corrected images for original Figure S1B and S1D.
