# Hypoxia induced exosomal circRNA promotes metastasis of Colorectal Cancer via targeting GEF-H1/RhoA axis: Erratum

**DOI:** 10.7150/thno.98415

**Published:** 2024-05-18

**Authors:** Haiou Yang, Haiyang Zhang, Yuchong Yang, Xinyi Wang, Ting Deng, Rui Liu, Tao Ning, Ming Bai, Hongli Li, Kegan Zhu, Jialu Li, Qian Fan, Guoguang Ying, Yi Ba

**Affiliations:** 1 Tianjin Medical University Cancer Institute and Hospital, National Clinical Research Center for Cancer, Key Laboratory of Cancer Prevention and Therapy, Tianjin's Clinical Research Center for Cancer, Tianjin, 300060, China.; 2 Division of Gastroenterology and Hepatology, Shanghai Institute of Digestive Disease, China.; 3 Key Laboratory of Gastroenterology and Hepatology, Ministry of Health, Shanghai Jiao-Tong University School of Medicine, Renji Hospital, China.

The original version of this article contains an error. In Figure 7A, the result of β-actin in HCT116 group was wrong pasted. We carried out a careful examination, and found that the error arose inadvertently as a consequence of multiple original pictures being opened simultaneously during the process of collating the data. The corrected figures do not affect the original conclusions of the findings. We sincerely apologize for any inconvenience this error may have caused.

The correct Figure 7A appears below.

## Figures and Tables

**Figure 7A:**
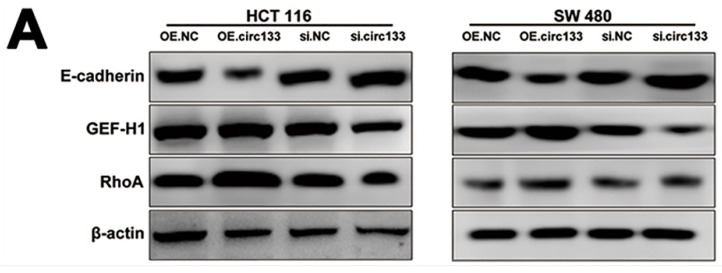
Correct image.

