# Supplementary material for: Hypoxia induced exosomal circRNA promotes metastasis of Colorectal Cancer via targeting GEF-H1/RhoA axis
Source: Theranostics. 2020 Jul 9;10(18):8211–26. doi: 10.7150/thno.44419 (PMC7381736; doi:10.7150/thno.44419)
Supplement: Supplementary file 1 — Supplementary figures and tables. [file thnov10p8211s1.pdf]

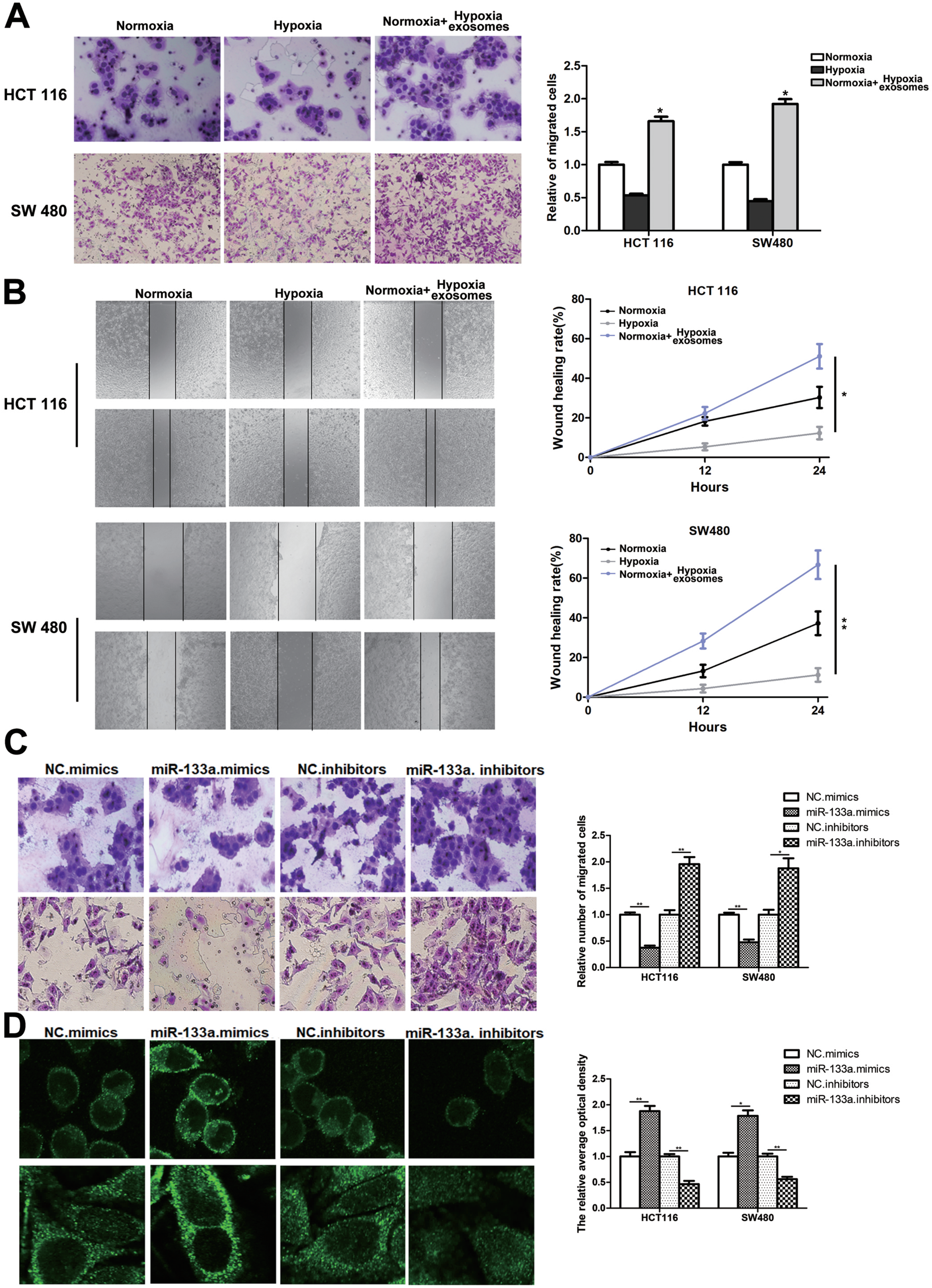

A
Normoxia
Hypoxia
Normoxia+ Hypoxia exosomes
HCT 116
SW 480
Relative of migrated cells
HCT 116
SW480
B
Normoxia
Hypoxia
Normoxia+ Hypoxia exosomes
HCT 116
SW 480
Wound healing rate(%)
Hours
SW480
C
NC.mimics
miR-133a.mimics
NC.inhibitors
miR-133a. inhibitors
miR-133a.inhibitors
Relative number of migrated cells
HCT116
SW480
D
NC.mimics
miR-133a.mimics
NC.inhibitors
miR-133a. inhibitors
miR-133a.inhibitors
The relative average optical density
HCT116
SW480

A

Characteristics	Number of patients (%) n=25
Gender	
Male	14(56%)
Female	11(44%)
Age	
＜60	18(72%)
≥60	7(28%)
Smoking history	
No	9(36%)
Yes	16(64%)
Drinking history	
No	6(24%)
Yes	19(76%)
Family History	
No	21(84%)
Yes	4(16%)
TNM classification	
I+ II	13(52%)
III+ IV	12(48%)

B

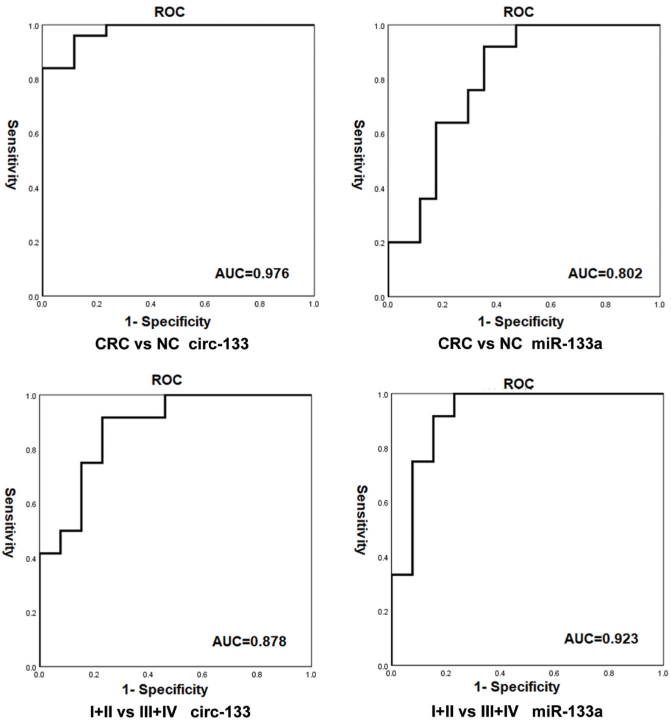

C

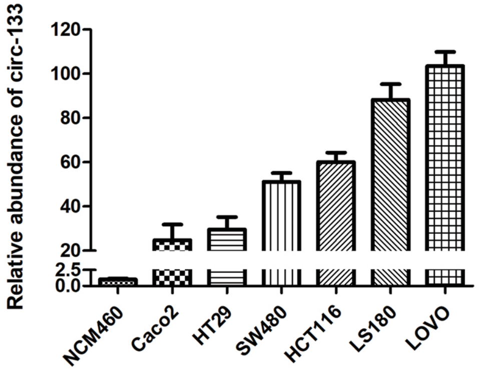

Fig.S1

(A) Validation of hypoxic exosomes enhance the migration ability of CRC cells by transwell assays, (n=3). (B) Wound healing assay demonstrated that hypoxic exosomes promote CRC cell migration, (n=3). (C) Transwell assays revealed that down-expression of miR-133a increase CRC cells migration, (n=3). (D) Immunofluorescence assay was used to detect E-cadherin membrane distribution in each treatment group, (n=3).

Fig.S2

(A) Basic clinical information of 25 CRC patients. (B) ROC curve according to the result of qRT-PCR. (C) Relative abundance of circ-133 in CRC cell lines, (n=3).
